# Calcium Sensors as Key Hubs in Plant Responses to Biotic and Abiotic Stresses

**DOI:** 10.3389/fpls.2016.00327

**Published:** 2016-03-16

**Authors:** Benoît Ranty, Didier Aldon, Valérie Cotelle, Jean-Philippe Galaud, Patrice Thuleau, Christian Mazars

**Affiliations:** Laboratoire de Recherche en Sciences Végétales, Université de Toulouse, CNRS, UPSAuzeville, Castanet-Tolosan, France

**Keywords:** CML, CPK, calcium signaling, specificity, calcium sensor, decoding, biotic and abiotic stress, plant

## Abstract

The Ca^2+^ ion is recognized as a crucial second messenger in signaling pathways coupling the perception of environmental stimuli to plant adaptive responses. Indeed, one of the earliest events following the perception of environmental changes (temperature, salt stress, drought, pathogen, or herbivore attack) is intracellular variation of free calcium concentrations. These calcium variations differ in their spatio-temporal characteristics (subcellular location, amplitude, kinetics) with the nature and strength of the stimulus and, for this reason, they are considered as signatures encrypting information from the initial stimulus. This information is believed to drive a specific response by decoding via calcium-binding proteins. Based on recent examples, we illustrate how individual calcium sensors from the calcium-dependent protein kinase and calmodulin-like protein families can integrate inputs from various environmental changes. Focusing on members of these two families, shown to be involved in plant responses to both abiotic and biotic stimuli, we discuss their role as key hubs and we put forward hypotheses explaining how they can drive the signaling pathways toward the appropriate plant responses.

## Introduction

Plants as sessile organisms have to continuously face environmental cues coming from abiotic and biotic challenges. Their survival depends on their ability to discriminate each stimulus among the diverse challenging environmental changes in order to prepare a specific response. Plants have evolved sophisticated signaling strategies allowing them, in most cases, to withstand these stresses and survive deleterious conditions. Among the strategies employed, plants use an intricate signaling network involving calcium as a second messenger. It is now well acknowledged that the Ca^2+^ ion plays a crucial role, as a mediator, in regulating and specifying the cellular responses to environmental stresses (Sanders et al., 2002; White and Broadley, 2003; Dodd et al., 2010). Each stimulus perceived by the plant is generally followed by an immediate increase in intracellular Ca^2+^ concentration occurring either simultaneously or after a lag time in a single or several intracellular compartments of the cell (cytosol, nucleus, mitochondria, etc.). Such Ca^2+^ transients, each linked to a particular stimulus, differ in their spatio-temporal patterns, being therefore considered as signatures (McAinsh and Hetherington, 1998). Each Ca^2+^ signature contributes to a first layer of specificity through its tissue and sub-cellular location, amplitude and frequency and the calcium pool involved in producing it (Allen et al., 2001; Miwa et al., 2006). However, to be fully significant in terms of signaling, decoding Ca^2+^ signals by Ca^2+^-modulated proteins (usually termed calcium sensors) is mandatory. Calcium sensor proteins estimated to number over 250 in *Arabidopsis* (Day et al., 2002) are represented by three main families, i.e., the calcineurin-B-like proteins (CBLs) (Luan, 2009), the calmodulin (CaM), and calmodulin-like proteins (CMLs) (Yang and Poovaiah, 2003; Bender and Snedden, 2013), the calcium-dependent protein kinases (CPKs) and the calcium and calmodulin-dependent protein kinase (CCaMK) (Cheng et al., 2002; Wang et al., 2015).

These proteins display various affinities for calcium ions and this property, combined with their sub-cellular location within the cell, will control their behavior. Calcium binding to Ca^2+^ sensors will induce a conformational change that triggers either their association to downstream target proteins or a direct stimulation of the kinase activity when CPKs are considered (Harmon et al., 2000). The diversity of Ca^2+^ sensors and their downstream targets contributes to a second layer of specificity, allowing the transduction of various primary stimuli into distinct biological responses (Hashimoto and Kudla, 2011). In addition, the hypothesis of signaling microdomains gathering calcium signaling components as described in animal cells (Good et al., 2011; Gueguinou et al., 2015) can also be proposed to contribute to response specificity in plants.

In this mini-review, we focus on calcium sensors lying at the crossroads of signaling pathways initiated by either abiotic or biotic stimuli. Due to the paucity of reported data concerning the role of the CBL/CIPK family in biotic stress responses, we will only consider CPK and CML protein families as common components of both biotic and abiotic stress signaling. We will discuss how a single Ca^2+^ sensor may direct the flow of signaling information toward distinct adaptive responses.

## CMLs at the Crossroads of Biotic and Abiotic Signaling Pathways

In addition to CaM, which has been well conserved through evolution, plant genomes are predicted to encode a broad range of CML proteins (McCormack et al., 2005; Boonburapong and Buaboocha, 2007; Zhu et al., 2015). Like CaM, most CMLs contain 4 EF-hand calcium-binding motifs and no other functional domains; they share at least 16% overall amino acid identity with CaM in *Arabidopsis* (McCormack and Braam, 2003). Several CMLs were shown to display biochemical properties typical of proteins that function as Ca^2+^ sensors, including, upon calcium binding, a shift in their electrophoretic mobility, and changes in secondary structure and in exposed surface hydrophobicity (Kohler and Neuhaus, 2000; Chiasson et al., 2005; Bender et al., 2014; Scholz et al., 2015). Thus, CMLs are believed to act as calcium sensors, and the Ca^2+^-induced conformational changes likely increase their interaction affinity to downstream effectors, as described for CaM. Search for CML targets has allowed the identification of diverse CML-binding proteins including protein kinases, transcription factors, metabolic enzymes, and transporters (Yamaguchi et al., 2005; Popescu et al., 2007; Perochon et al., 2010). Although regulation of these targets by CMLs is most often presumptive, CMLs likely function as calcium sensors/relays by tuning the activity of downstream effectors. Diverse roles for CMLs in plant development and stress responses have been reported, (Perochon et al., 2011; Bender and Snedden, 2013; Cheval et al., 2013; Zeng et al., 2015).

Our work on Ca^2+^-mediated stress signaling in the model plant *Arabidopsis*, has identified AtCML9 as both a positive and negative regulator of plant immune response and drought stress tolerance, respectively (Magnan et al., 2008; Leba et al., 2012). *AtCML9* was found to be induced early in plants exposed to salt, cold, or dehydration treatment, to a bacterial pathogen and to application of stress-associated phytohormones including abscisic acid (ABA) and salicylic acid (SA). Salt-responsive expression of *AtCML*9 is dependent on ABA production, while its expression in response to a virulent strain of *Pseudomonas syringae* is dependent on SA synthesis, suggesting that this CML is involved in stress hormone-mediated responses. In this respect, *Atcml9* null mutants exhibit a hypersensitive response to ABA during early growth stages that could be correlated with the enhanced tolerance to drought and salt stress in adult plants. *Atcml9* mutants also show alterations in the expression of several stress and ABA-responsive genes as well as defense-related genes. These data indicate that AtCML9 contributes to both abiotic and biotic stress responses in conjunction with hormone signaling, and suggest a role for AtCML9 in the regulation of stress/defense-related genes. AtCML9 was reported to interact with diverse transcription factors including WRKY53 and TGA3 two factors that participate in plant defense responses (Popescu et al., 2007). However, the biological relevance of these physical interactions requires further analysis.

Among other CMLs shown to be linked to stress signaling, AtCML37 and 42, appear to play dual roles in abiotic stress responses and defense against herbivorous insects. Loss of function of AtCML42 in *Arabidopsis* mutants results in enhanced resistance to *Spodoptera littoralis*, up-regulation of jasmonic acid (JA)-responsive genes and an increased accumulation of aliphatic glucosinolates (Vadassery et al., 2012). Thus, AtCML42 acts negatively on herbivore resistance by decreasing the expression of JA-responsive genes and the accumulation of defense secondary metabolites. *Atcml42* null mutants also show a reduced content in flavonol glucosides that play a role in UV-B protection. As a consequence, *Atcml42* mutant seedlings are less resistant to UV-B exposure. In contrast to AtCML42, AtCML37 acts positively on defense against *S. littoralis* (Scholz et al., 2014). AtCML37 loss-of-function mutants exhibit an enhanced susceptibility to herbivory correlated with a lower level of the bioactive form of jasmonate (JA-Ile), an important hormone in plant defense against herbivores, and lower expression of JA-responsive genes encoding proteins involved in the synthesis of molecules toxic for insects. In addition, *Atcml37* null mutants show a decrease in drought stress tolerance correlated with a low content in ABA, whereas *Atcml42* null mutants are not impaired in drought stress response (Scholz et al., 2015). Collectively, these data reveal opposite roles for AtCML37 and 42 in insect herbivory resistance and distinct functions of these two CMLs in abiotic stress responses. It can also be noted that these particular CMLs, at the crossroads of biotic and abiotic signaling pathways, play antagonistic roles for the plant, being generally protective in one pathway and deleterious in the second. Due to their dual role in distinct signaling pathways, these Ca^2+^ sensors are interesting tools that can help in understanding how specificity is achieved in plant responses to environmental cues. Identification of their interacting partners will be helpful to clarify how these CMLs exert their action at a molecular level and will shed light on the mechanisms controlling their antagonistic effects in defense against herbivores.

## CPKs as Signaling Nodes Mediating Plant Responses to Biotic and Abiotic Stresses

Calcium-dependent protein kinase and CMLs share a similar broad distribution in the plant kingdom (Valmonte et al., 2014; Zhu et al., 2015). They possess a CaM-like and a kinase domain that make them direct effectors upon activation by Ca^2+^binding. Their conserved molecular structure consists of a variable N-terminal domain joined to a Ser/Thr kinase domain associated to a CPK activation domain (CAD). CAD comprises an inhibitory junction domain (also termed pseudosubstrate region) fused to a CaM-like domain (Harper et al., 2004). The current *in vivo* activation model of CPKs states that, upon Ca^2+^ binding, the conformational change induced will release the pseudosubstrate region from the active site of the kinase domain (Liese and Romeis, 2013). Like CMLs, and according to their tissue and sub-cellular location or Ca^2+^ affinity, CPKs participate as Ca^2+^ sensors in the regulation of various plant functions ranging from plant growth and development to plant defense and adaptation against pathogens, pests, and environmental cues (Asano et al., 2012a; Boudsocq and Sheen, 2013; Schulz et al., 2013; Romeis and Herde, 2014). A number of studies have been performed to find substrates of CPKs (Rodriguez Milla et al., 2006; Uno et al., 2009; Mehlmer et al., 2010; Berendzen et al., 2012), but only a few have led to “*in vivo*” confirmation. Among them, the NADPH oxidase RBOHD, shown to be activated by AtCPK5 in *Arabidopsis* (Dubiella et al., 2013) is involved in ROS-mediated long distance signaling in response to several stimuli (Miller et al., 2009; Gilroy et al., 2014; Romeis and Herde, 2014). Other targets are located in guard cells where they display ion transport activity (Schroeder and Hagiwara, 1990; Pei et al., 1996; Brandt et al., 2012; Scherzer et al., 2012; Zhang et al., 2014). Interestingly, as for CMLs, some members of the CPK family appear to play a dual role in both biotic and abiotic stress responses.

In a recent study, aiming at understanding how calcium can regulate sphingolipid-induced programmed cell death (PCD), we identified AtCPK3 as a crucial effector in this process (Lachaud et al., 2013). Long chain bases (LCBs) which are precursors of more complex sphingolipids, have been proposed to mediate immune responses in plants (Peer et al., 2010). Some natural analogs mimicking LCBs, such as mycotoxins produced by necrotrophic *Fusarium* fungi, likely interfere with the sphingolipid signaling pathway and plant immunity processes. Indeed, fumonisin B1 (FB1) produced by *Fusarium monoliforme*, is able to quickly increase the level of free LCBs in the cell thus mimicking a LCB treatment (Saucedo-Garcia et al., 2011). In addition FB1 induces a Ca^2+^ burst that activates AtCPK3 which forms a complex with dimeric 14-3-3 proteins in resting conditions. Upon activation, AtCPK3 phosphorylates a conserved serine residue located at the N-terminal part of each 14-3-3 monomer. Phosphorylation of 14-3-3 proteins at the dimer interface results in disruption of their dimeric structure, promoting AtCPK3 release and cell death induction. Using *Atcpk3* null lines, we demonstrate that AtCPK3 is required for full development of PCD symptoms in leaves infiltrated with this mycotoxin (Lachaud et al., 2013). In parallel studies, using co-expression assays in *Nicotiana benthamiana*, AtCPK3 was also shown to be involved in biotic stress responses during plant-insect interactions. In wild-type plants exposed to *Spodoptera littoralis*, AtCPK3 is able to phosphorylate the heat-shock transcription factor B2a (HsfB2a) that likely activates the transcription of the defensin gene *PDF1.2*, known to be induced as a resistance mechanism (Kanchiswamy et al., 2010). Besides participation of AtCPK3 in signaling processes associated with biotic stresses, various studies also describe its involvement in the transduction of abiotic signals. Indeed, genetic approaches have shown that this kinase, together with AtCPK6, mediates ABA responses in guard cells. They participate in Ca^2+^ activation of plasma membrane slow-type anion channels (Mori et al., 2006). In the *Atcpk3/Atcpk6* double mutant, ABA-induced stomatal closure is impaired while long-term Ca^2+^-programmed stomatal closure is not. In addition, AtCPK3 binds and can phosphorylate the *Arabidopsis* vacuolar two-pore K^+^ channel TPK1, thereby contributing to its regulation during salt stress response (Latz et al., 2013) whereas during the same stress, AtCPK3 was shown to modulate the membrane phosphoproteome likely in a MAPK-independent pathway (Mehlmer et al., 2010).

Another example illustrating the dual role of CPKs in plant responses to biotic and abiotic stimuli concerns *AtCPK1*, which when over-expressed confers a SA-mediated resistance phenotype in *Arabidopsis* toward both necrotrophic fungi (*Botrytis cinerea, Fusarium oxysporum*) and biotrophic bacteria (*Pseudomonas syringae*), while its suppression (null mutants) results in an enhanced susceptibility to these pathogens (Coca and San Segundo, 2010). Like AtCPK3, AtCPK1 is thought to mediate responses to other environmental physical cues such as light. Using *Vicia faba* vacuoles and a patch-clamp approach, Schroeder’s group showed that recombinant AtCPK1 was able to activate a vacuolar chloride channel that could be, according to the authors, an important player in light-mediated stomatal opening (Pei et al., 1996).

Finally, in an elegant and exhaustive work, Boudsocq et al. (2010) identified several CPKs activated by flg22, a 22-amino acid peptide derived from flagellin known to induce plant immune responses (Boudsocq et al., 2010). By transiently expressing constitutively active *Arabidopsis* CPKs, five AtCPKs (AtCPK4,5,6,11,26) were found able to activate the expression of the Ca^2+^-dependent flg22-responsive reporter gene NHL10-LUC (NDR1/Hin1-*Like10*-LUCiferase), in the same manner as flg22 (Boudsocq et al., 2010). In the light of the presumed functions of AtCPKs (Boudsocq and Sheen, 2013), it is interesting to note that the AtCPKs mentioned above play a dual role being also involved in abiotic signaling pathways. AtCPK4 and AtCPK11 were shown to promote the expression of the reporter luciferase (LUC) under the control of an ABA-responsive promoter (*RD29A)*. The activity of LUC was synergistically increased upon co-expression of AtCPK4 with the ABA-responsive bZIP transcription factor ABF2, whereas AtCPK11 which shares more than 95% identity with AtCPK4 was unable to enhance the LUC response through ABF2 (Lu et al., 2013).

## How Can a Single Calcium Sensor Contribute Specific Responses to Both Biotic and Abiotic Stresses

**FIGURE 1:**
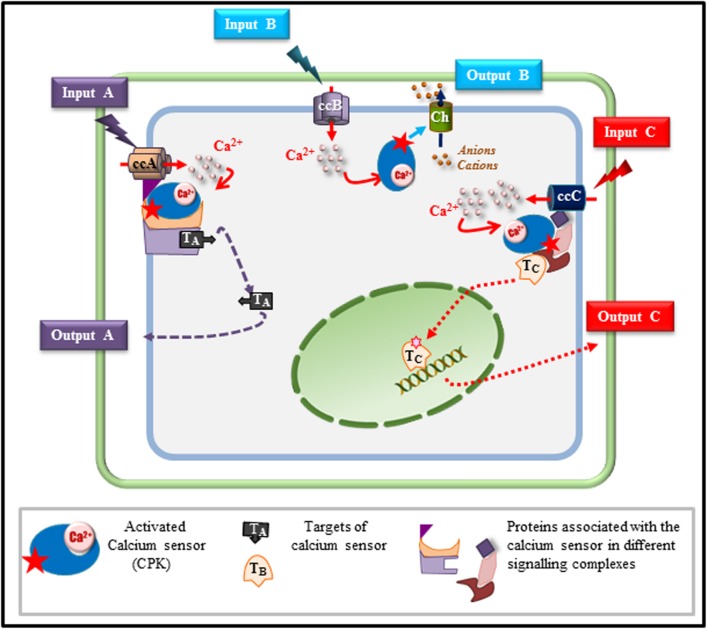
**Model depicting how a single calcium sensor can handle various cell signaling inputs and outputs**. Based on the results described in this mini-review, we depict various possible scenarios explaining how a single calcium sensor such as a calcium-dependent protein kinase (CPK), can drive signaling pathways toward specific responses in a single cell exposed to different stimuli. In response to a stimulus A (input A), a specific calcium channel (ccA) is activated and generates a calcium flux that will activate the CPK that will phosphorylate target A (TA) involved in output A. In response to a second stimulus (input B), a specific calcium channel is activated (ccB) and calcium released into the cytosol will activate the CPK that can directly regulate an effector (Ch) such as an ion transporter (channel, pump, exchanger) leading, according to the charge of the transported ions, to membrane depolarisation or hyperpolarisation contributing to the final response (output B). The same calcium sensor can be associated, in the same cell, with a different scaffold tethered to a different calcium channel (ccC) that is activated only by stimulus C (input C). Upon ccC activation, the calcium sensor is activated and can lead, through this specific signaling module, to the activation of a different target (T_C_) migrating to the nucleus in order to control the adaptive response C through gene reprogramming for instance (output C).

From the different examples described above, it appears that CPKs as CMLs, share the property of being hubs, able to integrate signals triggered by both biotic and abiotic stimuli and to drive the signaling pathway toward the appropriate response. This property that seems common to related Ca^2+^ sensors in other plants species, including monocots (Asano et al., 2012b), raises the question of the mechanisms involved in the switching functions that these CPKs or CMLs are expected to play. While it is quite difficult to answer this question from studies performed on CMLs, mainly due to our lack of knowledge concerning their downstream targets, different hypotheses can be drawn from existing knowledge on CPKs (**Figure 1**). Taking AtCPK3, as a model, we can speculate on the way in which a single Ca^2+^ sensor can wire the flow of signaling information toward the right adaptive response upon challenging the plant with different stimuli. Beyond the importance of the specific Ca^2+^ signature generated in response to a given stimulus, it is clear that specificity of the routing will be achieved only if the calcium sensor, its downstream targets and putative partner proteins are co-localized and expressed at the same time. In guard cells, AtCPK3 can be activated by calcium ions flowing through non-selective calcium channels in response to environmental cues (e.g., drought) (Schroeder and Hagiwara, 1990) and can phosphorylate S-type anion channels resulting in stomatal closure. When located in mesophyll cells, the situation is more complex if we consider that these cells can be challenged by different stimuli (e.g., herbivores and mycotoxins) leading to different outputs (e.g., defense and cell death). Exposure of these cells to the chewing action of *S. littoralis* is reported to stimulate glutamate receptors (Mousavi et al., 2013) that have been shown to mediate Ca^2+^ influx (Kwaaitaal et al., 2011; Manzoor et al., 2013). These Ca^2+^ fluxes in mesophyll cells will probably account for AtCPK3 activation which in turn will phosphorylate HsfB2a, a transcription factor up-regulating the defensin gene involved in responses against herbivores (Kanchiswamy et al., 2010). However, if glutamate channels can be suspected in the case of *S. littoralis* attack, they are most probably not involved in the cytosolic Ca^2+^ transients observed in response to FB1/LCBs. Indeed, ionotropic glutamate channel inhibitors did not inhibit the cytosolic Ca^2+^ responses when cells were challenged with LCBs (Lachaud et al., 2010). A different type of Ca^2+^ channel could be involved in FB1-induced calcium increase leading to PCD. Therefore, in this particular case, the switching mode of AtCPK3, leading either to PCD or basal defense mechanisms, can rather be related to the origin of Ca^2+^ and/or the nature of the channels involved in shaping the Ca^2+^ signatures triggered by each stimulus (insect or sphingolipid). It can then be hypothesized that these different classes of activated channels can engage AtCPK3 in different signaling modules that are specifically associated to each type of channel. Such an association will favor the interaction of AtCPK3 with its downstream substrates specifically dedicated to one output (defense or cell death). Similar assemblies of signaling proteins defined as signaling complexes or scaffolds have been shown for a few signaling proteins in plants. Indeed AtCPK21 was shown to interact in an ABA-dependent manner with the slow anion channel 1 (SLAC1) homolog 3 (SLAH3) in microdomains of the guard cell plasma membrane. The activity of AtCPK21, which is crucial for SLAH3 activation, seems to be regulated by a protein scaffold involving protein phosphatase 2C (PP2C) ABI1 and an ABA receptor of the RCAR1/PYR/PYL family (Demir et al., 2013). This scaffold hypothesis has got a much larger scientific audience in animals (Good et al., 2011) where clustering of Ca^2+^ channels in membrane microdomains and association with their downstream signaling complexes have been reported (Dart, 2010; Gueguinou et al., 2015).

## Concluding Remarks

The operating mode of these signaling hubs remains a challenging question that needs further investigation. Emerging imaging techniques at nanoscale resolution (Curthoys et al., 2015; Komis et al., 2015) and new proteomics approaches dedicated to deciphering protein complexes (Boeri Erba, 2014; Liu and Heck, 2015; Politis and Borysik, 2015) are very promising and should lead to a better understanding of the mechanisms underlying how a single Ca^2+^ sensor acts at the crossroads of various signaling pathways to help plants to cope with various environmental challenges.

## Author Contributions

CM and BR contributed to the writing of the manuscript. DA and CM participated in drawing **Figure 1** and in critically revising the manuscript with PT, J-PG, and VC.

## Conflict of Interest Statement

The authors declare that the research was conducted in the absence of any commercial or financial relationships that could be construed as a potential conflict of interest.
